# Diabetes mellitus im Kindes- und Jugendalter (Update 2023)

**DOI:** 10.1007/s00508-023-02169-5

**Published:** 2023-04-20

**Authors:** Birgit Rami-Merhar, Elke Fröhlich-Reiterer, Sabine E. Hofer, Maria Fritsch

**Affiliations:** 1 Universitätsklinik für Kinder- und Jugendheilkunde, Medizinische Universität Wien, Währinger Gürtel 18–20, 1090 Wien, Österreich; 2 Universitätsklinik für Kinder- und Jugendheilkunde, Abteilung für allgemeine Pädiatrie, Medizinische Universität Graz, Graz, Österreich; 3 Universitätsklinik für Kinder- und Jugendheilkunde, Department für Pädiatrie, Medizinische Universität Innsbruck, Innsbruck, Österreich

**Keywords:** Typ 1 Diabetes, Metabolische Kontrolle, Schwere Hyperglykämien, Insulinpumpentherapie, Type 1 diabetes, Metabolic control, Severe hypoglycemic events, Insulin pump treatment

## Abstract

Im Kindes- und Jugendalter ist, im Gegensatz zum Erwachsenenalter, der Diabetes mellitus Typ 1 (T1D) die am häufigsten auftretende Form des Diabetes mellitus (> 90 %). Nach der Diagnosestellung sollte die Betreuung der Kinder und Jugendlichen in einem pädiatrischen Zentrum mit viel Erfahrung in pädiatrischer Diabetologie erfolgen. Eine lebenslange Insulintherapie ist notwendig, wobei diese individuell an das Alter und den Alltag der Familie angepasst werden soll. In diesem Alter wird ausdrücklich die Verwendung von Diabetestechnologie (Sensorglukosemessung, Insulinpumpentherapie und seit kurzem eine Hybrid-Closed-Loop-Therapie) empfohlen. Eine möglichst optimale metabolische Einstellung ab Therapiebeginn verbessert die Langzeitprognose der jungen Menschen mit Diabetes. Ein wesentlicher Teil in der Betreuung ist die Schulung von PatientInnen und Eltern von einem entsprechend ausgebildeten multidisziplinären Team, bestehend aus pädiatrischen DiabetologInnen, DiabetesberaterInnen, DiätologInnen, PsychologInnen und SozialarbeiterInnen. Die APEDÖ (Arbeitsgruppe für pädiatrische Endokrinologie und Diabetologie Österreich) und die ISPAD (International Society for Pediatric and Adolescent Diabetes) empfehlen als metabolisches Ziel für alle pädiatrischen Altersgruppen einen HbA_1c_-Wert ≤ 7,0 % (IFCC ≤ 53 mmol/mol) mit einer „Time in Range“ (TIR) > 70 % ohne schwere Hypoglykämien. Eine altersentsprechend normale körperliche, kognitive und psychosoziale Entwicklung sowie die Vermeidung von Akutkomplikationen (schwere Hypoglykämien, diabetische Ketoazidose), das Screening auf assoziierte Erkrankungen und die Prävention von diabetesbedingten Spätkomplikationen zum Erhalt einer hohen Lebensqualität sind die Ziele der pädiatrischen Diabetestherapie.

## Abstract (en)

In contrast to adults, type 1 diabetes mellitus (T1D) is the most frequent form of diabetes in childhood and adolescence (> 90%). After diagnosis the management of children and adolescents with T1D should take place in highly specialized pediatric units experienced in pediatric diabetology. The lifelong substitution of insulin is the cornerstone of treatment whereby modalities need to be individually adapted for patient age and the family routine. In this age group the usage of diabetes technology (glucose sensors, insulinpumps and recently hybrid-closed-loop-systems) is recommended. An optimal metabolic control right from the start of therapy is associated with an improved long-term prognosis. Diabetes education is essential in the management of patients with diabetes and their families and needs to be performed by a multidisciplinary team consisting of a pediatric diabetologists, diabetes educator, dietitian, psychologist and social worker. The Austrian working group for pediatric endocrinology and diabetes (APEDÖ) and the ISPAD (International Society for Pediatric and Adolescent Diabetes) recommend a metabolic goal of HbA1c ≤ 7.0%, ((IFCC) < 53 mmol/mol), and a “Time in range” > 70% for all pediatric age groups without the presence of severe hypoglycemia. Age-related physical, cognitive and psychosocial development, screening for associated diseases, avoidance of acute diabetes-related complications (severe hypoglycemia, diabetic ketoacidosis) and prevention of diabetes-related late complications to ensure high quality of life are the main goals of diabetes treatment in all pediatric age groups.

## Definition

Diabetes mellitus ist eine Stoffwechselerkrankung mit unterschiedlicher Ätiologie, welche durch eine persistierende Hyperglykämie, bedingt durch eine Störung der Insulinsekretion und/oder Insulinwirkung charakterisiert ist.

## Klassifikation

Die derzeit gültige Klassifikation der American Diabetes Association (ADA 2022) und der ISPAD 2022 teilt die verschiedenen Diabetesformen in Typ 1–4 ein [1, 2]. Im Kindes- und Jugendalter tritt in Österreich zu > 90 % ein Diabetes mellitus Typ 1 (T1D) auf, der aufgrund des Insulinmangels rasch zu einer diabetischen Ketoazidose (DKA) führen kann. In den letzten Jahren konnte in Österreich ein signifikanter Anstieg der DKA bei Erstmanifestation beobachtet werden. Die bedeutet, dass die Diagnose oft verzögert und sehr spät gestellt wird [3, 4]. Die Erstmanifestation eines T1D kann in jedem Kindes- und Jugendalter auftreten, auch im Säuglingsalter, der Erkrankungsgipfel liegt im Volksschulalter, es sind aber zunehmend jüngere Kinder betroffen.

Weitere im Kindes- und Jugendalter vorkommende Diabetesformen sind Diabetes mellitus Typ 2 (T2D), spezifische Diabetes-mellitus-Typen (z. B. MODY (Maturity Onset Diabetes of the Young), CF-related DM (CFRD), nach Transplantation, bei Kortisontherapie) sowie assoziiert bei verschiedenen Syndromen (z. B. Trisomie 21, Prader-Willi-Syndrom u. a.).

## Epidemiologie

Der T1D ist die häufigste Stoffwechselerkrankung im Kindes- und Jugendalter. Die Inzidenz der Erkrankung < 15 Jahren nimmt weltweit kontinuierlich zu [3, 5, 6].

Die aktuellsten Daten des österreichischen Diabetes-Inzidenz-Registers in der Altersgruppe 0 bis 14 Jahre stammen aus dem Zeitraum 1989–2021 [3]. Von den 5888 in diesem Zeitraum erfassten Fälle hatten 94,3 % einen T1D, 1,8 % einen T2D und 3,9 % eine andere Form des Diabetes. Nachdem in den Jahren 1989–2011 ein kontinuierlicher Anstieg der T1D Inzidenz beobachtet wurde (annual percent change [APC] 4,57 %, *p* < 0,001) folgte in den Jahren 2012–2020 eine Plateauphase (APC 0,78 %, *p* = 0,379). Im Jahr 2021 wurde mit 28,6/100.000/Jahr (95 % CI: 25,7–31,6) die höchste österreichische standardisierte T1D Inzidenzrate beobachtet. Diese Zunahme im Jahr 2021 fällt zeitlich, mit der globalen Covid-19-Pandemie zusammen. Im Inzidenz-Register sind keine Infektionen oder Impfungen erfasst, daher kann kein kausaler Zusammenhang gezogen werden.

Zum ersten Mal konnte ein statistisch signifikanter Anstieg der T2D-Inzidenzrate beobachtet werden (1999–2021: APC 3,47 %, *p* = 0,014). Andere Diabetesformen kommen in der Altersgruppe bis < 15 Jahren aber doppelt so oft vor wie der T2D.

## Klinische Symptome

Beim T1D im Kindes- und Jugendalter treten meist klassische Symptome wie Polyurie, Enuresis, Polydipsie, Gewichtsverlust, Müdigkeit, Konzentrationsstörungen, Sehstörungen, Verhaltensauffälligkeiten oder Soorinfektionen auf, wobei die Dauer dieser Symptome meist kurz (Tage bis Wochen) ist. Wird die Diagnose spät gestellt, dann treten auch Symptome einer diabetischen Ketoazidose auf (u. a. vertiefte Atmung, Übelkeit, Erbrechen, Bewusstseinstrübung). Je jünger das Kind ist, desto schwieriger kann es sein, die Symptome einer Diabetesmanifestation zuzuordnen.

## Diagnosekriterien für einen Diabetes mellitus

Die Diagnose eines T1D im Kindesalter wird meist anhand der typischen Symptome, einer Harnuntersuchung, der Blutzucker- und HbA_1c_-Bestimmung gestellt, zusätzlich sind bei rund 85 % der Kinder und Jugendlichen mit T1D diabetesspezifische Autoantikörper (ICA, IA2, IAA, GAD, ZnT8-Ak) nachweisbar. Es gelten in der Pädiatrie die gleichen Diagnosekriterien wie bei Erwachsenen, lediglich die Glukosebelastung beim oralen Glukosetoleranztest (oGTT) ist gewichtsbedingt unterschiedlich. Ein oGTT ist bei Kindern und Jugendlichen mit T1D nur selten notwendig, spielt aber bei der Diagnose seltenerer Diabetesformen (z. B. T2D, MODY, CF-related DM) eine wichtige Rolle (Tab. 1).

**Table 1:** 

HbA_1c_ > 6,5 % (IFCC > 48 mmol/mol) (DCCT-standardisiertes Labor)
oder
Nüchtern-Plasma-Glukose ≥ 126 mg/dl (mindestens 8 h keine Kalorienaufnahme)
oder
2‑h-Plasma-Glukose beim oGTT ≥ 200 mg/dl (der oGTT soll mit einer Glukosebelastung von 1,75 g/kgKG, maximal 75 g durchgeführt werden)
oder
Klassische diabetesspezifische Symptome oder hyperglykämische Krise mit einer Plasmaglukose ≥ 200 mg/dl

Bei klassischen Symptomen und Hyperglykämie und/oder Glukosurie/Ketonurie sollten die Kinder/Jugendlichen umgehend an eine Kinderabteilung mit ausreichend Erfahrung in der Behandlung von Kindern mit Diabetes und Expertise in pädiatrischer Diabetologie überwiesen werden. Der Anstieg der DKA-Rate bei Erstmanifestation weist auf eine verzögerte Diagnosestellung hin. Fast jedes 2. Kind mit Diabetesmanifestation wird in Österreich zu spät diagnostiziert [4].

Bei Kindern und Jugendlichen mit fehlender klinischer Symptomatik, aber nachgewiesener Hyperglykämie und/oder Glukosurie (dies kann z. B. transient im Rahmen eines Infektes auftreten) sollten eine Kontaktaufnahme sowie eine weitere Abklärung in einem Zentrum für pädiatrische Diabetologie erfolgen.

## Therapie und Ziele

Die Betreuung und Behandlung von Kindern und Jugendlichen mit Diabetes mellitus sollte grundsätzlich in einem Zentrum für pädiatrische Diabetologie bzw. einer Kinderabteilung mit ausreichender Erfahrung in pädiatrischer Diabetologie erfolgen. Diese Zentren benötigen eine ausreichende personelle Besetzung mit einem multidisziplinärem Team [7].

### Ziele

Eine altersentsprechend normale körperliche, kognitive und psychosoziale Entwicklung, sowie die Vermeidung von Akutkomplikationen (schwere Hypoglykämien, diabetische Ketoazidose) und die Prävention von diabetesbedingten Spätkomplikationen (diabetische Retinopathie, diabetische Nephropathie, diabetische Neuropathie u. a.) zum Erhalt einer hohen Lebensqualität sind die Ziele der pädiatrischen Diabetestherapie.

Dies beinhaltet eine regelmäßige Kontrolle des Längen- und Gewichtsverlaufs, des Pubertätsstatus sowie die Durchführung regelmäßiger Screening-Untersuchungen auf chronische Folgeerkrankungen im Frühstadium und diabetesassoziierte Erkrankungen (Zöliakie, Schilddrüsenerkrankungen u. a.).

Die österreichische Arbeitsgruppe für pädiatrische Endokrinologie und Diabetologie (APEDÖ), wie auch die ISPAD (International Society for Pediatric and Adolescent Diabetes) empfehlen als Ziel für die metabolische Einstellung HbA_1c_-Werte ≤ 7,0 % (IFCC ≤ 53 mmol/mol) [8, 9]. Die Empfehlungen internationaler Diabetesgesellschaften reichen von HbA_1c_ ≤ 6,5 % (≤ 48 mmol/mol, NICE – [10]) bis ≤ 7,0 % (≤ 53 mmol/mol, ISPAD – [9]), auch die ADA hat erst kürzlich das Ziel auf ≤ 7,0 % (≤ 53 mmol/mol), gesenkt [11]. Es gilt, den niedrigsten HbA_1c_-Wert anzustreben, welcher ohne schwere Hypoglykämien zu erreichen ist. Mit der Verwendung der Sensorglukosewerte werden inzwischen vermehrt diese zur Beurteilung der metabolischen Einstellung herangezogen, weil sie im Gegensatz zum HbA_1c_ (Durchschnittswerte ohne Information zu Hypo- und Hyperglykämien) einen besseren Einblick in die glykämische Variabilität geben. Der Begriff „Time in Range“ (TIR = Zeit im Zielbereich), d. h. der Prozentsatz an Zeit mit Sensorglukosewerten im Bereich zwischen 70–180 mg/dl, hat in den letzten Jahren zunehmend an Bedeutung gewonnen und ist zu einer neuen Messgröße zusätzlich zum HbA_1c_ geworden. Mehr als 70 % eines Tages sollten in diesem Bereich verbracht werden. Weitere Ziele sind weniger als 4 % des Tages unter 70 mg/dl (3,9 mmol/l), weniger als 1 % unter 54 mg/dl (3 mmol/l) und < 25 % > 180 mg/dl (10 mmol/l), bzw. < 5 % > 250 mg/dl (13,9 mmol/l) zu verbringen [12]. Die Zeit im Zielbereich korreliert mit dem HbA_1c_-Wert hinsichtlich des Folgeerkrankungsrisikos [13].

### Kontinuierliche Behandlung bei Diabetes mellitus Typ 1

#### Insulintherapie

Eine Insulintherapie muss ab dem Zeitpunkt der Diagnosestellung eingeleitet und lebenslang fortgesetzt werden, wobei der Insulinbedarf in unterschiedlichen Phasen der Erkrankung variieren kann. Es wird empfohlen bei Kindern und Jugendlichen eine individualisierte, intensivierte Insulintherapie einzuleiten, dafür stehen folgende Therapien zur Verfügung: Basis-Bolustherapie, Insulinpumpentherapie, sensorunterstützte Pumpentherapie, Hybrid-Closed-Loop-Systeme.

Zur prandialen Insulinsubstitution und zur Korrektur sollten kurzwirksame Insuline, schnell oder ultraschnell wirksame Insulinanaloga, welche sich hinsichtlich ihres Wirkbeginns und der Wirkdauer unterscheiden, verwendet werden. In Insulinpumpen sollten vorzugsweise schnell oder ultraschnell wirksame Insulinanaloga Verwendung finden [14]. Bei Kleinkindern ist gelegentlich aufgrund des geringen Insulinbedarfs die Verwendung von verdünntem Insulin (U10/U20/U50) notwendig. Die Verdünnung kann mittels physiologischer Kochsalzlösung oder mit, von der pharmazeutischen Industrie, zur Verfügung gestelltem Verdünnungsmedium hergestellt werden. Die Haltbarkeit der verdünnten Insuline ist begrenzt (4 Wochen). Die Verdünnung selbst sollte vorzugsweise von Apotheken hergestellt werden.

Als Basalinsuline werden überwiegend lang oder ultralang wirksame Insulinanaloga (seltener NPH-Insuline) zur Substitution des basalen Insulinbedarfs verwendet. Lang wirksame Insuline finden bei der Pumpentherapie keine Verwendung, da der Basalbedarf durch die kontinuierliche Abgabe von schnell wirksamen Insulinanaloga (Basalrate) abgedeckt wird.

Die Insulinpumpentherapie sollte allen Kindern und Jugendlichen jeglichen Alters empfohlen werden [15, 16], bei folgenden Indikationen aber jedenfalls eingesetzt werden

- Kleinkinder (auch Säuglinge), Kinder und Jugendliche mit ausgeprägtem Dawn-Phänomen, Risiko für Hypoglykämien, rezidivierende Hypoglykämien, fehlende Hypoglykämiewahrnehmung, nächtlichen Hypoglykämien, Nadelphobie, hohe glykämische Variabilität unabhängig vom HbA_1c_, bei Vorliegen von diabetischen Spätschäden (Retinopathie, Nephropathie, Neuropathie), schwangere Jugendliche.

Die Hybrid-Closed-Loop-Therapie ist eine neue Technologie, bestehend aus drei Komponenten:

- *Glukosesensor:* Der Glukosesensor, der im subkutanen Fettgewebe die Glukosewerte alle paar Minuten – kontinuierlich – misst.
- *Algorithmus:* Diese gemessenen Glukosewerte werden direkt an eine Insulinpumpe oder eine Handy-App gesendet. Der jeweilige Algorithmus, berechnet automatisch wieviel Insulin der Körper benötigt um die Glukosewerte im Zielbereich zu halten.
- *Insulinpumpe:* Das automatisierte Algorithmus-System, kommuniziert mit der Pumpe und die entsprechend berechnete Insulinmenge wird über die Insulinpumpe ins subkutane Fettgewebe abgegeben.

Alle derzeit in Österreich erhältlichen und im Kindesalter zugelassenen Systeme (ab 1. Lebensjahr CamDiab, ab 7. Lebensjahr Medtronic 780G) sind Hybrid-Closed-Loop-Systeme, das bedeutet, dass für die Mahlzeiten Boli abgegeben werden müssen. Die Anwendung dieser Systeme geht mit einer Steigerung der Zeit im Zielbereich und einer Reduktion des HbA_1c_, ohne Steigerung von Hypoglykämien einher [17–19].

Eine gute metabolische Einstellung von Beginn an ist prognostisch essentiell [20–22], daher ist es notwendig, eine individualisierte, altersadäquate Therapie anzuwenden, um eine hohe Therapiezufriedenheit und ein gutes metabolisches Outcome zu erreichen.

#### Glukosemessung

Eine regelmäßige Glukoseselbstmessung entweder durch Blutzuckerbestimmung in kapillarem Blut aus der Fingerbeere oder mittels kontinuierlicher subkutaner Glukosemessung (isCGM oder rtCGM) muss begleitend zur Insulintherapie bei allen Kindern durchgeführt werden. Die blutige Messung der Glukosewerte wurde in den vergangenen Jahren weitestgehend durch subkutane, kontinuierliche Glukosemessungen verdrängt. Bei alleiniger Blutzuckermessung mittels Kapillarblut ist eine Häufigkeit von 6 bis 10 Messungen täglich empfohlen. Die Messung sollte jeweils VOR den Mahlzeiten und in der Nacht stattfinden. Bei Anwendung von Glukosesensoren können blutige Messungen zur Kalibration oder zur Kontrolle bei nicht plausiblen Werten empfohlen sein.

Glukosesensoren mit subkutaner kontinuierlicher Glukosemessung sind für alle Altersgruppen ab dem 1. Lebensjahr anwendbar. Die Liegedauer unterscheidet sich je nach Gerät. Eine regelmäßige Dokumentation der Glukosewerte, Insulindosen und Kohlenhydrateinheiten erscheint im Alltag hilfreich und kann zunehmend auch mit elektronischen Hilfsmitteln erfolgen. Alle diabetesrelevanten Geräte (Pumpe, Glukosemessgeräte, Sensoren) sollten im Rahmen der ärztlichen Kontrolle ausgelesen, standardisiert ausgewertet und analysiert werden, um therapeutische Maßnahmen ableiten und empfehlen zu können.

Weitere Details und Empfehlungen siehe ÖDG Leitlinie Technologie.

### Therapie bei Diabetes mellitus Typ 2 (T2D)

Zur Therapie des T2D wird eine Lebensstilmodifikation in Kombination mit einer medikamentösen Therapie empfohlen [23, 24]. Für eine medikamentöse Therapie des T2D sind derzeit Metformin (ab dem 10. Lebensjahr), Insulin und seit 2019 auch das GLP-1-Analogon Liraglutid (ab dem 10. Lebensjahr) zugelassen [23, 24]. Weitere neuere Medikamente wie DPP-4-Inhibitoren oder SGLT-2-Inhibitoren, sind für die pädiatrische Altersgruppe noch nicht erprobt bzw. zugelassen.

Als Ziel für die metabolische Einstellung wird auch beim T2D ein HbA_1c_-Wert ≤ 7,0 % (IFCC ≤ 53 mmol/mol) angestrebt.

**Figure 1:**
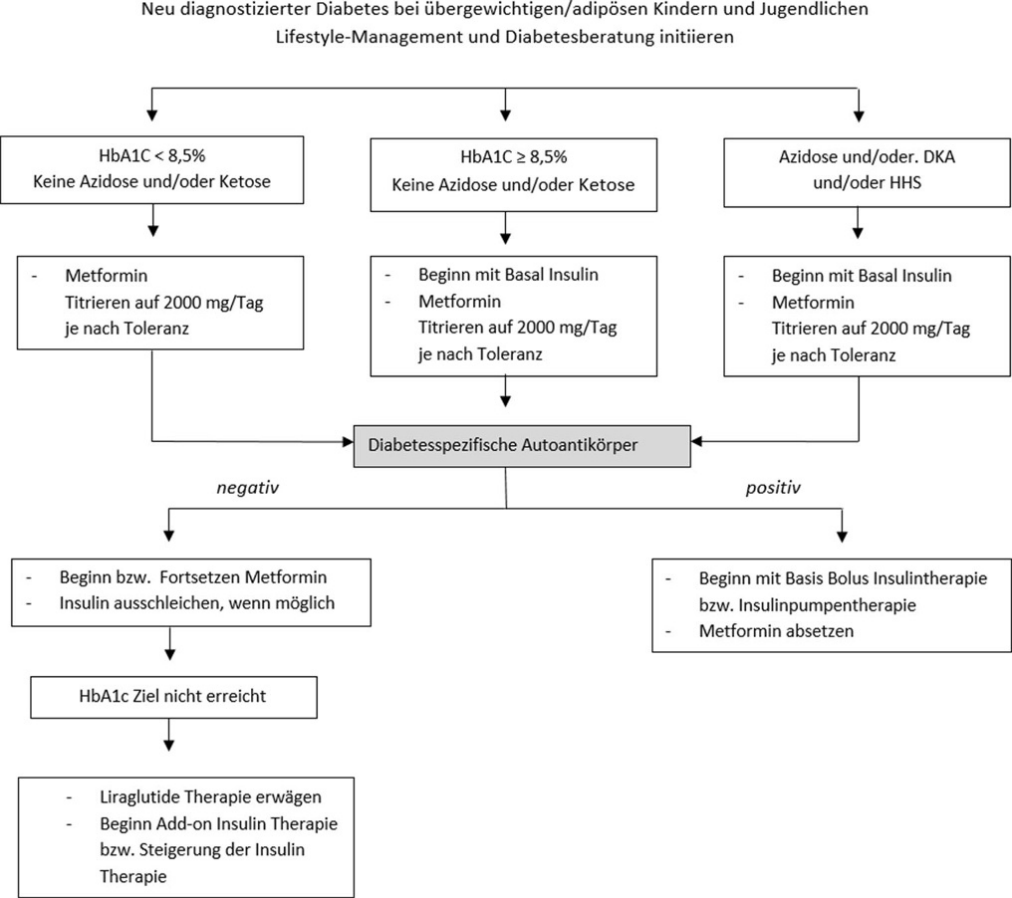

Die initiale Behandlung sollte von klinischer Symptomatik, Schweregrad der Hyperglykämie bzw. Bestehen einer Ketose/Ketoazidose abhängig gemacht werden ([23, 24]; Abb. 1).

1. Bei metabolisch stabilen, asymptomatischen Patienten mit einem HbA_1c_-Wert < 8,5 % (69,4 mmol/mol) soll initial mit einer Monotherapie mit Metformin begonnen werden. Falls das HbA1c Ziel ≤ 7,0 % (≤ 53 mmol/mol) unter maximal empfohlener Metformindosis nicht erreicht wird, soll die Erweiterung der Therapie um Liraglutid bzw. Insulin erwogen werden [24], wobei der Vorteil einer Therapie mit Liraglutid im Vergleich zu Insulin im positiven Effekt auf den Gewichtsverlauf liegt. Einschränkend für die Therapie mit Liraglutid kann allerdings das häufige Auftreten von gastrointestinalen Nebenwirkungen sein.
2. Bei Patienten mit einem HbA_1c_-Wert ≥ 8,5 % (69,4 mmol/mol) oder Vorliegen einer Ketose/Ketonurie oder Ketoazidose muss initial mit einer Insulintherapie begonnen werden. Zu beachten ist allerdings, dass eine Insulintherapie mit dem Risiko einer unerwünschten Gewichtszunahme und, wenn auch selten, mit Hypoglykämien einhergehen kann.
  Bei Patienten ohne Ketoazidose kann zeitgleich eine Therapie mit Metformin eingeleitet werden [24].
3. Bei Ausschleichens der Insulintherapie sollte, auch wenn keine diabetesspezifischen Auto-Antikörper nachgewiesen werden konnten, an die Möglichkeit des Vorliegens eines T1D gedacht werden, insbesondere wenn bei der Diabetesdiagnosestellung eine DKA vorlag.

#### Ernährung

Die Ernährungsschulung und das Einhalten einer kohlenhydratberechnenden Kost sind eine wichtige Grundvoraussetzung für eine gute metabolische Einstellung. Die Schulung über die Berechnung der Nahrung (insbesondere der Kohlenhydrate) und deren Wirkung auf den Blutzucker sollte von DiätologInnen durchgeführt werden. Es sollte dabei auf kulturelle Ernährungsgewohnheiten Rücksicht genommen werden.

Neben einer altersgerechten Kalorien- und Energiezufuhr muss ein ausreichender Anteil an Kohlenhydraten (50–55 %) zur Sicherstellung eines regulären altersentsprechenden Wachstums zugeführt werden [25].

Der prandiale Insulinbedarf wird durch die Berechnung der Kohlehydrate ermittelt. Kohlehydrate werden im deutschsprachigen Raum als Broteinheit (BE = 12 g Kohlehydrate) oder Kohlehydrateinheit (KE = 10 g Kohlehydraten) angegeben. Bei neueren Pumpenmodellen erfolgt die Angabe allerdings in Gramm Kohlehydraten.

Neben der Berechnung der Kohlehydrate zeigt die Berücksichtigung des glykämischen Index (GI) mit Bevorzugung von Kohlehydraten mit niedrigen GI positive Effekte auf die glykämische Kontrolle [25].

#### Schulung

Eine altersangepasste, strukturierte Diabetesschulung ist integrativer Bestandteil der therapeutischen Bemühungen und Voraussetzung für ein funktionierendes Diabetesmanagement zu Hause. Die Diabetesschulung umfasst alle in dieser Behandlungsempfehlung aufgelisteten Aspekte der pädiatrischen Diabetestherapie. Eltern bzw. Betreuer aus dem sozialem Umfeld müssen ins Diabetesmanagement eingebunden werden.

Das multidisziplinäre Schulungs-Team sollte pädiatrischen DiabetologInnen, DiabetesberaterInnen, DiätologInnen, PsychologInnen sowie SozialarbeiterInnen umfassen.

#### Psychologische Betreuung

Das multidisziplinäre Behandlungsteam sollte durch PsychologInnen unterstützt werden, welche bei der Erfassung der psychosozialen Situation der PatientInnen/Familien im Rahmen dieser chronischen Erkrankung eine wichtige Rolle einnehmen und ggf. auch spezifische Interventionen durchführen können. Der Erstkontakt mit Kind und Familie erfolgt idealerweise bei Diabetes Erstmanifestation.

Zahlreiche psychiatrische Komorbiditäten sind bei T1D bekannt (Essstörungen, Insulinmanipulation, Depression, Angststörung, ADHS u. a.).

## Akute Komplikationen

Zu den Akutkomplikationen des T1D zählen zum einen die schwere Hypoglykämie und zum anderen die diabetische Ketoazidose (DKA).

Die schwere Hypoglykämie ist definiert als ein Ereignis mit Bewusstseinsbeeinträchtigung (Koma oder Krampfanfall), das einer Fremdhilfe bedarf.

Die Behandlung der schweren Hypoglykämie erfolgt im Homesetting mit Glukagon Nasenspray (3 mg nasal ab dem 4. Lebensjahr) oder Glukagon Fertigspritze (0,5–1 mg) i.m. oder s.c. (keine Altersbeschränkung). Im Kliniksetting kann eine Hypoglykämie auch mit intravenöser Glukosezufuhr von 2–3 ml/kgKG 10 % Glukose erfolgen. In jedem Haushalt mit einem Kind mit T1D sollte die Notfallmedikation Glukagon (Nasenspray oder Glukagon Fertigspritze) vorhanden sein. Die Behandlung mit Glukagon setzt eine entsprechende Schulung voraus [26].

Kleinkinder sind aufgrund der mangelnden Fähigkeit, Hypoglykämiesymptome zu äußern, und durch eine verminderte Hypoglykämiewahrnehmung einem höheren Risiko für das Auftreten einer schweren Hypoglykämie ausgesetzt.

Internationale Registerstudien konnten zeigen, dass die Inzidenz der schweren Hypoglykämien in den letzten zwei Dekaden abgenommen hat. Eine Korrelation von niedrigen HbA_1c_-Werten und damit verbunden höherer Hypoglykämierate konnte nicht bestätigt werden [15].

Eine weitere akute Komplikation ist die diabetische Ketoazidose (DKA). Wie bereits erwähnt, wurde in Österreich im Zeitraum 2012 bis 2020 ein deutlicher Anstieg der DKA bei Diabetesmanifestation beobachtet, wobei bei 44 % der Kinder und Jugendlichen im Rahmen der Diabetesmanifestation eine Ketoazidose (schwere DKA) festgestellt wurde [4]. Auch im Verlauf der Diabeteserkrankung ist eine DKA möglich, die Ursache ist immer Insulinmangel [27]. Die klinischen Zeichen einer DKA sind: Dehydratation, Tachykardie, Tachypnoe, Kußmaul-Atmung, Azetongeruch, Übelkeit und Erbrechen, Schläfrigkeit bis hin zum Koma. Risikofaktoren für eine DKA bei Erstmanifestation sind: jüngeres Alter, verzögerte Diagnosestellung, niedriger sozioökonomischer Status und Länder mit niedriger Diabetesinzidenz. Risikofaktoren für eine DKA bei bekanntem T1D sind v. a. absichtliches Weglassen der Insulininjektionen, Fehlverhalten bei Krankheit und Fehlverhalten bei Insulinpumpentherapie, sowie das Vorliegen von psychiatrischen Vorerkrankungen (inkl. Essstörungen).

Die Therapie und Überwachung von Kindern mit DKA sollte von ÄrztInnen durchgeführt werden, die über Erfahrung in diesem Gebiet verfügen, und es muss die Möglichkeit zu einer intensivmedizinischen ärztlichen, pflegerischen und biochemischen Überwachung gesichert sein.

Die Therapieziele der DKA sind: Ausgleich der Dehydratation, Ausgleich der Azidose, Blutzuckerstabilisierung und -normalisierung und Vermeidung von Komplikationen (Hirnödem und Hypokaliämie). Die Therapie besteht aus Flüssigkeits‑, Elektrolyt- (Kalium) und Insulinsubstitution (über 24–48 h). Mit einer iv-Flüssigkeitssubstitution sollte unverzüglich, noch vor Beginn der Insulin- und Elektrolytsubstitution, in Form von 0,9 % NaCl-Lösung begonnen werden. Vor Beginn der Kaliumsubstitution sollte die Diurese bzw. Nierenfunktion gesichert werden [27].

Die Vermeidung von akuten Komplikationen zählt zu den vorrangigen Zielen in der Betreuung von Kindern und Jugendlichen mit T1D.

## Langzeitkomplikationen und Screening-Untersuchungen

Bei den Follow-up-Untersuchungen sollen routinemäßig die Körperlänge, das Körpergewicht, der Blutdruck (unter Verwendung von alters- und geschlechtsspezifischen Perzentilen und BMI-Standards), das Pubertätsstadium (Tanner Stadien) sowie die Injektionsstellen bzw. Katheter-Injektionsstellen (cave Lipohypertrophien/Lipoatrophien und Hautirritationen) und der CGM-Stellen (Ekzeme/Hautirritationen/Abszesse) kontrolliert werden [28, 29].

Einmal im Jahr ist auch die Kontrolle der Nieren- und Leberfunktionsparameter, des Blutbildes sowie des Lipidstatus indiziert. Der HbA_1c_ sollte alle 3 Monate bestimmt werden.

Um das Risiko für mikrovaskuläre und makrovaskuläre Komplikationen zu senken, sollte eine möglichst normoglykämische Stoffwechseleinstellung angestrebt werden, um das Auftreten von diabetischen Komplikationen, wie eine diabetische Retinopathie, eine Mikroalbuminurie, eine Hypertonie oder eine diabetische Neuropathie bei Jugendlichen zu verhindern [30].

Ein jährliches Screening auf mikrovaskuläre Komplikationen wie Nephropathie (Morgenharn: Albumin/Kreatinin-Ratio), Retinopathie (Funduskopie) und Neuropathie (klinische Untersuchung auf Sensibilität, Vibrationsempfinden und Reflexe) wird jährlich ab dem 11. Lebensjahr oder 2 bis 5 Jahren Diabetesdauer empfohlen [30].

Ein Screening auf makrovaskuläre Komplikationen (Lipidstatus) sollte kurz nach Diagnosestellung (nach Stabilisierung der Stoffwechsellage) bei allen Kindern, die älter als 11 Jahre sind, durchgeführt werden. Sind die Blutfette normal, genügen 3‑jährige Screening-Untersuchungen nach dem 11. Lebensjahr. Der RR sollte mindestens 1 × jährlich, idealerweise bei jeder Visite kontrolliert werden [30].

Bei Verdacht auf eine Hypertonie sollte eine 24-h-Blutdruckmessung durchgeführt werden (unter Verwendung von alters- und geschlechtsspezifischen Normwerten) [30]. Zusätzlich zu einer Lifestyleintervention (DASH-Diät, Steigerung der körperlichen Aktivität und Reduktion von sitzenden Tätigkeiten), werden ACE-Hemmer zur Senkung eines erhöhten Blutdrucks im Kindes- und Jugendalter empfohlen und haben sich als sichere und effektive Therapie in Kurzeitstudien erwiesen. Der klinische Benefit von Angiotensin-II-Rezeptorantagonisten ist ähnlich wie bei ACE-Hemmern, wobei ihre Verwendung in der Kinder- und Jugendheilkunde nicht so weit verbreitet ist. Aufgrund des teratogenen Potentials sollte vor Beginn einer ACE-Hemmer Therapie bei Mädchen eine ausführliche Aufklärung erfolgen und eine effektive Verhütungsmethode implementiert werden. Des Weiteren vermindern ACE-Hemmer die Progression von einer Mikroalbuminurie zur Makroalbuminurie und erhöhen die Regressionsrate zur Normoalbuminurie [30, 31]. Bei Vorliegen einer Hypercholesterinämie haben Kurzzeitstudien gezeigt, dass Simvastatin und Pravastatin bei Kindern effektiv und sicher in der Therapie der Hypercholesterinämie sind [30]. Es wurden keine signifikanten Nebenwirkungen im Hinblick auf Wachstum, Pubertätsfortschritt, endokrine Funktion sowie Leber und Muskelenzyme beobachtet, jedoch sollte ein Augenmerk auf Symptome gelegt werden, die die Leber sowie die Muskeln oder das Bindegewebe betreffen, da das Risiko einer Rhabdomyolyse bei Anwendung von Statinen erhöht ist. Bei Statinen muss aufgrund der potenziell teratogenen Wirkung bei Mädchen eine ausführliche Aufklärung erfolgen und ggf. eine effektive Verhütungsmethode implementiert werden [30, 31].

Bei an T2D erkrankten Patienten sollte bereits zum Diagnosezeitpunkt ein vollständiges Screening auf mikro- und makrovaskuläre Komplikationen erfolgen [30].

## Assoziierte Erkrankungen

Kinder und Jugendliche mit T1D haben ein höheres Risiko, weitere Autoimmunerkrankungen zu entwickeln. Die häufigsten assoziierten Autoimmunerkrankungen sind die Autoimmunthyreoiditis (AIT) und die Zöliakie (CD).

Bis zu 29 % der Menschen mit T1D haben bei T1D Erstmanifestation positive Schilddrüsenantikörper, welche prädiktiv für die Entwicklung einer AIT sind, 3–8 % davon entwickeln eine AIT wobei die Meisten eine Hypothyreose haben. Die AIT ist die häufigste assoziierte Autoimmunerkrankung und kommt häufiger bei Mädchen vor, oft manifestiert sie sich in der Pubertät, und sie ist mit einer längeren Diabetesdauer assoziiert [28, 29].

Ein Screening auf assoziierte AIT (mittels TSH, fT4, fT3 und TPO-^−^und Tg-AK) wird bei Erstmanifestation (nach klinischer Stabilisierung) und danach alle 2 Jahre bei asymptomatischen Patienten empfohlen. Bei Vorliegen einer Struma, schlechter Wachstumsgeschwindigkeit oder klinischen Symptomen einer Schilddrüsen-Funktionsstörung sollte früher gescreent werden.

Die Hyperthyreose kommt, bei Kindern und Jugendlichen mit T1D, deutlich seltener vor als die Hypothyreose, jedoch häufiger als in der Normalbevölkerung. Die Prävalenz wird mit 0,5–6 % angegeben.

Die Prävalenz der Zöliakie (CD) wird bei Kindern und Jugendlichen mit T1D mit 1–16,4 % angegeben, wobei Kinder mit sehr jungem Diabetesmanifestationsalter ein höheres Risiko für die Entwicklung einer Zöliakie haben. Die klassischen Symptome der Zöliakie, wie z. B. Gedeihstörung, oder gastrointestinale Symptome sind eher selten, meist sind die Patienten asymptomatisch.

Ein Screening auf Zöliakie sollte mittels tTG IgA und/oder EMA und zusätzlichem Ausschluss eines IgA-Mangels durchgeführt werden. Bei nachgewiesenen IgA Mangel ist das Zöliakie-Screening mittels IgG basierten Tests (tTG IgG und/oder EMA IgG) empfohlen. Das Screening auf Zöliakie wird im ersten Jahr der Diagnosestellung und danach nach 2 Jahren und 5 Jahren empfohlen. Bei klinischen Symptomen oder erst-gradigen Verwandten mit Zöliakie sollte öfter gescreent werden. HLA-DQ2 und HLA-DQ8 sind bei Patienten mit T1D häufig positiv und werden deshalb nicht als Screening Untersuchung empfohlen.

Kinder mit positiven CD-Antikörpern sollten einem pädiatrischen Gastroenterologen vorgestellt werden. Die aktuellen ESPGHAN Guidelines aus 2020 empfehlen, dass bei Patienten mit hochpositiven tTG-AK (> 10 fache des oberen Normbereichs) auf eine Dünndarmbiopsie verzichtet werden kann [32]. In diesen neuen Guidelines wird aber nicht speziell auf die Gruppe der Kinder und Jugendlichen mit T1D eingegangen.

Die APEDÖ empfiehlt derzeit bei asymptomatischen Kindern mit T1D und positiven tTG-AK (unabhängig von der Höhe der AK) weiterhin die Duodenalbiopsie, um anhand der Marsh Klassifikation die Diagnose zu bestätigen [28]. Bei Kindern mit Symptomen und hochpositiven tTG-AK (> 10 fache des oberen Normbereichs) und positiven EMA kann in Absprache mit den pädiatrischen Gastroenterologen und der Familie eventuell auf eine Biopsie verzichtet werden [28, 29].

Weitere Autoimmunerkrankungen wie, Morbus Addison, rheumatoide Arthritis, Autoimmungastritis, chronisch entzündliche Darmerkrankungen, Vitiligo oder Polyendokrinopathien sind seltener. Bei Symptomen sollten DiabetologInnen aber auch an die Möglichkeit dieser selteneren Autoimmunerkrankungen denken.

## Transition

Im Alter von 18 bis 19 Jahren bzw. mit Abschluss der Schulausbildung/Lehre sollten die jungen Erwachsenen mit Diabetes zur langfristigen Betreuung durch Internisten an Abteilungen für Erwachsenenmedizin übergeben werden. Diese Transition soll flexibel, je nach „Reife“ des Jugendlichen, geordnet und im Idealfall im Rahmen einer Transitionsklinik erfolgen. Die Transition sollte möglichst frühzeitig besprochen und individuell geplant werden, und es sollte keine „Lücke“ in der Betreuung entstehen. Mit der Vorbereitung soll mindesten 1 Jahr vor Transfer begonnen werden. Besonderer Fokus soll auf Selfmanagement und Selbstverantwortung gelegt werden. Eine geschriebene Zusammenfassung (aktuelle Problemliste, Medikation, Stoffwechselkontrolle …) soll vorliegen. Spezielle Transitionskliniken und internistische Kliniken, die auf die Bedürfnisse der Jugendlichen eingehen und bei denen eine Verbindung zwischen pädiatrischem und internistischem Zentrum besteht, haben sich bewährt [33]. Rückmeldungen nach erfolgreicher Transition an den Pädiater sind wünschenswert.

## Interessenkonflikt

B. Rami-Merhar hat in den letzten 36 Monaten von folgenden Firmen Honorare/Forschungsgelder erhalten: Eli Lilly, Medtronic, Insulet, Abbott, Sanofi. E. Fröhlich-Reiterer hat in den letzten 36 Monaten von folgenden Firmen Vortragshonorare, Forschungsgelder und Honorare für Beratungstätigkeit erhalten: Medtronic, Novo Nordisk, Eli Lilly, Sanofi und Merck. S.E. Hofer erklärt in den letzten 3 Jahren Vortragshonorare der Firmen E. Lilly, Medtronic, Insulet, Dexcom und Ypsomed erhalten zu haben. M. Fritsch gibt an, dass kein Interessenkonflikt besteht.
